# A clinical scoring system for pediatric hand-foot-mouth disease

**DOI:** 10.1186/s12879-021-06424-w

**Published:** 2021-07-31

**Authors:** Hui Huang, Li Deng, Liping Jia, Runan Zhu

**Affiliations:** Department of Infectious Diseases, The Affiliated Children’s Hospital, Capital Institute of Pediatrics, Beijing, China

**Keywords:** Infants, Hand-foot-mouth disease, Enterovirus, Clinical diagnosis, Scoring system, Receiver operating characteristic curve

## Abstract

**Background:**

The aim of the present study was to develop a clinical scoring system for the diagnosis of hand-foot-mouth disease (HFMD) with improved accuracy.

**Methods:**

A retrospective analysis was performed on standardized patient history and clinical examination data obtained from 1435 pediatric patients under the age of three years who presented with acute rash illness and underwent enterovirus nucleic acid detection. Patients were then divided into the HFMD (1094 patients) group or non-HFMD (341 patients) group based on a positive or a negative result from the assay, respectively. We then divided the data into a training set (1004 cases, 70%) and a test set (431 cases, 30%) using a random number method. Multivariate logistic regression was performed on 15 clinical variables (e.g. age, exposure history, number of rash spots in a single body region) to identify variables highly predictive of a positive diagnosis in the training set. Using the variables with high impact on the diagnostic accuracy, we generated a scoring system for predicting HFMD and subsequently evaluated this system in the test set by receiver operating characteristic curve (ROC curve).

**Results:**

Using the logistic model, we identified seven clinical variables (age, exposure history, and rash density at specific regions of the body) to be included into the scoring system. The final scores ranged from − 5 to 24 (higher scores positively predicted HFMD diagnosis). Through our training set, a cutoff score of 7 resulted in a sensitivity of 0.76 and specificity of 0.68. The area under the receiver operating characteristic curve (AUC) was 0.804 (95% confidence interval [CI]: 0.773–0.835) (*P* < 0.001). Using the test set, we obtained an AUC of 0.76 (95% CI: 0.710–0.810) with a sensitivity of 0.76 and a specificity of 0.62. These results from the test set were consistent with those from the training set.

**Conclusions:**

This study establishes an objective scoring system for the diagnosis of typical and atypical HFMD using measures accessible through routine clinical encounters. Due to the accuracy and sensitivity achieved by this scoring system, it can be employed as a rapid, low-cost method for establishing diagnoses in children with acute rash illness.

## Background

Acute infectious rashes are among the most frequent presenting signs in the pediatric population. Commonly associated pathogens include enterovirus, measles virus, varicella zoster virus, rubella virus, and group A beta-hemolytic streptococcus. Hand-foot-mouth disease (HFMD) is among the most common of acute rashes caused by enterovirus infection [1]. The potential pathogens causing HFMD include enterovirus 71 [2], coxsackievirus group A 4 (CA4) [3], CA6 [4–6], CA10 [5, 7], and CAl6 [1–6]. While the majority of childhood HFMD patients present with classical features such as maculopapular rash, blisters, and/or ulcers in the mouth, hands, feet, and buttocks [8], HFMD can also exhibit unusual cutaneous manifestations that may be difficult to differentiate from other viral exanthemas [9–11]. For this reason, increased accuracy in diagnosing atypical HFMD will improve triage, treatment, and isolation of affected patients. The gold standard in the diagnosis of HFMD is the Polymerase Chain Reaction *(*PCR)-based viral nucleic acid sequence detection assay [12]. However, health care settings lacking this resource must continue to rely on clinical markers of the disease. In this retrospective study of more than 1400 children with acute rash illness, we analyzed multiple clinical variables to devise a scoring system that relies on elements that can be obtained during a routine patient encounter. To date, no studies have systematically investigated or identified clinical variables predictive of HFMD.

## Methods

We performed a retrospective analysis of patients who presented with acute rash illness to the Department of Infectious Diseases at the Capital Institute of Pediatrics Affiliated Children’s Hospital between January 2013 and December 2017. Prior to this period, clinicians were trained to complete an acute-rash-illness observation form, which collected information including patient age, gender, date of illness onset, exposure history, fever duration, and rash distribution and density. For rash quantification, the number of ulcers/sores in the oral cavity was rated as few (1–3 spots) or many (≥ 4) while the degree of rash was rated as low for 1–5 spots per body part and high for more than five spots per body part. Exposure history was defined as a close contact with individuals with confirmed HFMD or herpetic angina no more than 10 days prior to onset [13]. The inclusion criteria were as follows: (1) manifestation of an acute rash, (2) onset of illness of less than three days, (3) age of three years or less, and (4) completed the enterovirus throat-swab nucleic acid detection test. Patients were excluded if they had a definitive diagnosis of measles, rubella, or chickenpox.

Definitive diagnoses in all cases were established using enterovirus nucleic acid detection testing performed via throat swabs [14]. Total RNA was extracted from all specimens and the ABI7500 real-time fluorescence quantitative PCR system was then used for enterovirus nucleic acid detection.

Data analysis was performed using the SAS 9.4 software package (Windows, SAS Institute, Cary, North Carolina). Continuous variables distributed normally are expressed as mean ± standard deviation. Comparisons between the two groups were made using the independent t-test. Categorical variables were compared with the Chi-square test. A random number method was used to select 70% (1004) of cases as the training set for quantifying each clinical variable’s impact on a scoring model. The remaining 30% (431) of cases were used as the test set to evaluate the scoring model. Multivariate logistic regression analysis of clinical variables associated with HFMD was performed using stepwise regression to identify explanatory variables. Diagnostic HFMD scores were constructed using the Framingham study multi-factor model [15]. In this study, the ß value was divided by a constant B = 0.262 to obtain an integer value. The performance of the scoring system was assessed by calculating the area under the receiver operating characteristic (ROC) curve (AUC) as follows: 0.5–0.7 represented low diagnostic value, 0.7–0.9 represented intermediate diagnostic value, and > 0.9 represented high diagnostic value. Statistical significance was defined as *P* < 0.05. Our study protocol was reviewed and approved by the Capital Institute of Pediatrics Ethics Committee (SHERLL2019012). The data used in our study was desensitized, with unique codes replacing individual names, and the mailing addresses and contact information of the subjects were all replaced by codes. The individual could not be located based on database information.

## Results

A total of 1435 (823 males) patients were included in this study, where 1094 patients tested positive (HFMD group) for enterovirus RNA while 341 patients (non-HFMD group) tested negative (Table 1). Among 1094 confirmed cases of HFMD, 215 were EV710-positive, 283 were CA16-positive, and 596 were positive when tested with a set of non-EV71, non-CA16 universal primers for enterovirus (the pathogens include CA6, CA10, CA4, and other types of enterovirus). While no difference in gender composition was found between the two groups, HFMD patients were older and had longer illness duration when compared with non-HFMD patients (Table 1).

**Table 1:** Comparison of general and epidemiological data between the hand-foot-mouth disease (HFMD) and control groups

	Number of patients	Number of male patients	Illness duration (days)	Age (years)	Number of patients with positive exposure history
HFMD group	1094	634	1.68 ± 1.01	1.72 ± 0.76	442
Non-HFMD group	341	189	1.49 ± 0.88	1.51 ± 0.77	39
*χ*^*2*^*/t*		0.679	3.357	4.441	97.878
*P*		0.410	< 0.001	< 0.001	< 0.001

A subset of children in both groups (442 in the HFMD group and 39 in the non-HFMD group) endorsed a history of close contact with patients with HFMD or herpangina. The proportion of patients with clear exposure history was higher in the HFMD group than in the non-HFMD group (Table 1).

Since HFMD rashes are often concentrated in specific locations of the body, we quantified the rash severity by dividing the body into discrete regions. The oral cavity was divided into the hard palate, soft palate, tongue, buccal mucosa, lip mucosa, and gums. The remainder of the body was divided into the face, chest, back, buttocks, upper limbs, lower limbs, palms, back of the hands, fingers, feet, dorsum of the feet, plantar surface of the feet, and toes. In each patient, the number of rash spots/ulcers/sores in each body region was counted. We observed significant differences in rash densities in the upper jaw, soft palate, tongue, buccal mucosa, gums, chest, back, buttocks, and toes (Table 2).

**Table 2:** Rash distribution across body regions in hand-foot-mouth disease (HFMD) and non-HFMD patients

Body part	Hard palate			Soft palate			Tongue			Buccal mucosa			Lip mucosa			Gums		
Rash count	None	Few	Many	None	Few	Many	None	Few	Many	None	Few	Many	None	Few	Many	None	Few	Many
HFMD group	483	297	314	453	251	390	908	133	53	701	209	184	978	73	43	958	68	68
Non-HFMD group	229	72	40	210	68	63	317	16	8	277	47	17	318	15	8	317	11	13
*χ*^*2*^ */ t*	61.766			48.248			20.794			41.324			4.457			7.733		
*P*	< 0.001			< 0.001			< 0.001			< 0.001			0.11			0.021		
Body part	Face			Chest			Back			Buttocks			Upper extremities			Fewer extremities		
Rash count	None	Few	Many	None	Few	Many	None	Few	Many	None	Few	Many	None	Few	Many	None	Few	Many
HFMD group	1041	21	32	936	72	86	923	80	91	404	274	416	843	109	142	739	150	205
Non-HFMD group	316	14	11	253	30	58	246	33	62	192	74	75	265	34	42	241	48	52
*χ*^*2*^ */ t*	5.342			27.527			30.341			44.226			0.104			2.164		
*P*	0.07			< 0.001			< 0.001			< 0.001			0.95			0.34		
Body part	Palm			Back of hand			Fingers			Foot- plantar surface			Foot- dorsal surface			Foot-digits		
Rash count	None	Few	Many	None	Few	Many	None	Few	Many	None	Few	Many	None	Few	Many	None	Few	Many
HFMD group	260	446	388	749	218	127	583	305	206	494	351	249	781	189	124	596	290	208
Non-HFMD group	99	136	106	239	59	43	200	94	47	149	113	79	248	47	46	240	68	33
*χ*^*2*^ */ t*	4.382			1.247			5.134			0.232			3.013			29.268		
*P*	0.11			0.54			0.07			0.89			0.22			< 0.01		

Additionally, we analyzed additional clinical information such as fever severity, length of fever, fever-to-rash interval, presence of cough, gastrointestinal symptoms, WBC count, and neutrophil percentage. Between-group differences were found in fever frequency, WBC count, and neutrophil percentage (Table 3).

**Table 3:** Additional clinical variables

	Fever	Fever to rash duration (days)	Low fever (37–38 °C)	Intermediate fever (38–39 °C)	High fever (> 39)	Presence of cough	Gastrointestinal symptoms	WBC count (×10^9^/L)	Neutrophil percentage (%)
HFMD group (1094 cases)	756	0.58 ± 0.88	158	388	210	61	22	10.61 ± 3.65	49.2 ± 15.8
Non-HFMD group (341 cases)	199	0.64 ± 1.14	47	93	59	17	10	9.44 ± 3.52	39.3 ± 15.6
*χ*^*2*^ */ t*	13.487	−0.69	1.397			0.645	1.013	5.212	10.166
*P*	< 0.001	0.49	0.50			0.42	0.34	< 0.001	< 0.001

*HFMD* hand-foot-mouth disease

**Fig. 1:**
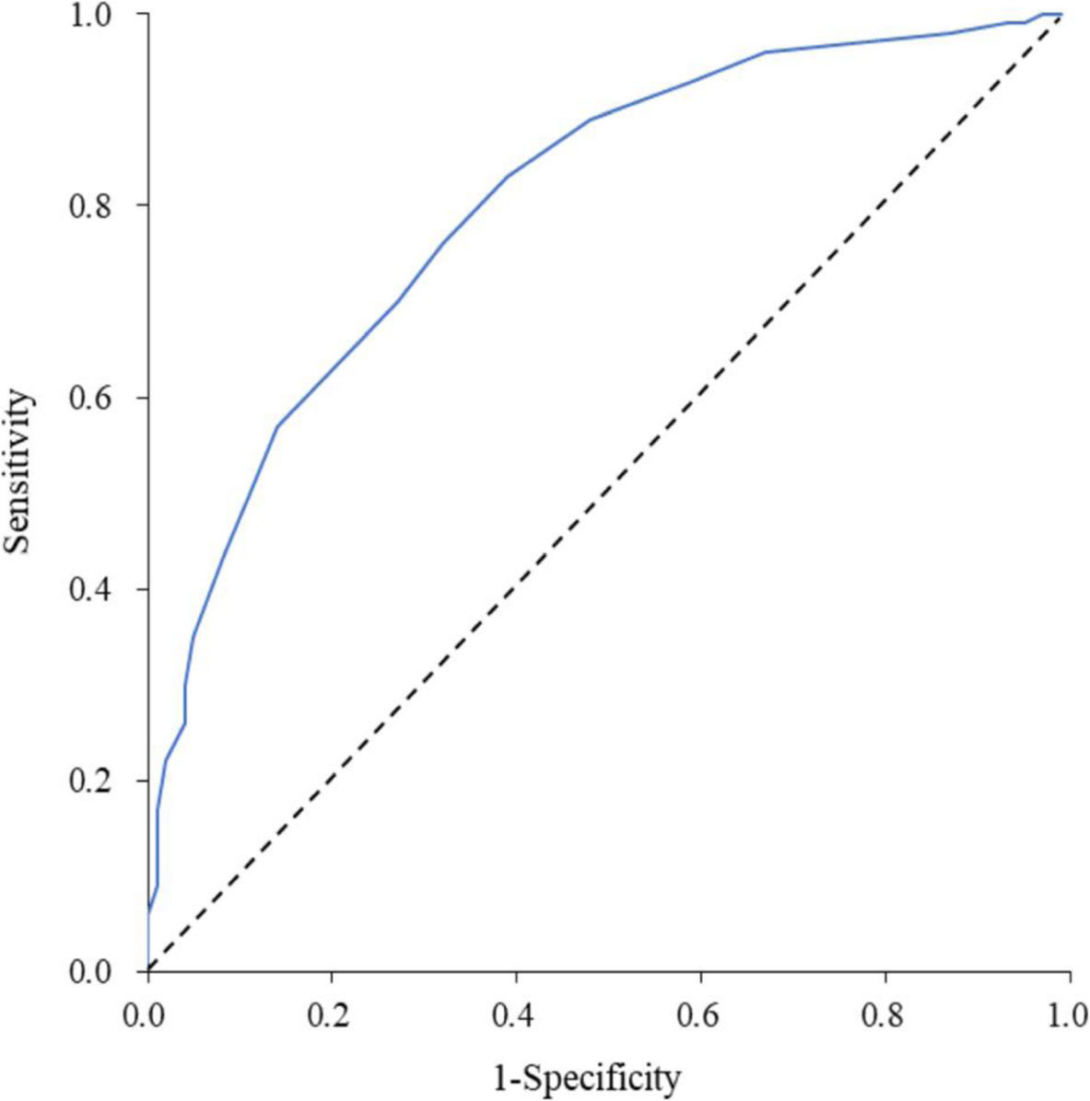
Area under the receiver operating characteristic curve for the scoring system (training set)

Of the 1004 cases in the training set, 764 were HFMD patients and 240 were non-HFMD patients. Multivariate logistic regression was then used to establish a scoring model based on the training set data. Among 15 tested clinical variables, 7 statistically significant clinical variables were identified and subsequently included in the scoring model. These included the following: (1) age; (2) exposure history; the number of ulcers on the (3) hard palate, (4) soft palate, (5) buccal mucosa; and cutaneous rash distributed on the (6) back and (7) buttocks (Table 4). To test the predictive accuracy of this scoring system, we applied this model on data from patients in the training set. The median score of the HFMD group was 10 (7, 15). The median score of the non-HFMD group was 4 (2, 8)**,** which was significantly lower than that in the HFMD group (Wilcoxon rank sum test, Z = 14.24, *P* < 0.001). The final scores ranged from − 5 to 24 points with predictive accuracies of 0.12 to 0.99. The AUC was 0.804 (95% confidence interval [CI]: 0.773–0.835), which was significantly higher than 0.5 (defined as having a predictive ability at chance; P < 0.001). The sensitivity was 0.76 and specificity was 0.68. Additionally, we found the positive predictive value to be 0.88 and the negative predictive value to be 0.47 (Fig. 1). We found the optimal cut-off point to be seven; hence, a score of seven or greater suggested a positive HFMD diagnosis, while a score of less than seven could be diagnosed as non-HFMD.

**Table 4:** Model established based on the training set and the scoring

Clinical variable		β	*P*	Odds ratio	95% confidence interval		Score
					Lower	Upper	
Age (for each additional 1 year of age)		0.26	0.0127	1.291	1.06	1.58	1
Exposure history		1.66	<.0001	5.269	3.35	8.30	7
Rash count							
Hard palate	Few	0.77	0.0003	2.151	1.43	3.24	3
	Many	0.75	0.0017	2.123	1.33	3.40	3
Soft palate	Few	0.54	0.0132	1.716	1.12	2.63	2
	Many	0.91	<.0001	2.48	1.62	3.80	4
Buccal mucosa	Few	0.48	0.0438	1.617	1.01	2.58	2
	Many	0.84	0.021	2.319	1.14	4.74	3
Back	Few	−0.39	0.212	0.68	0.37	1.25	−2
	Many	−1.33	<.0001	0.266	0.16	0.45	−5
Buttocks	Few	0.80	0.0002	2.228	1.46	3.39	3
	Many	1.34	<.0001	3.823	2.48	5.88	5

**Fig. 2:**
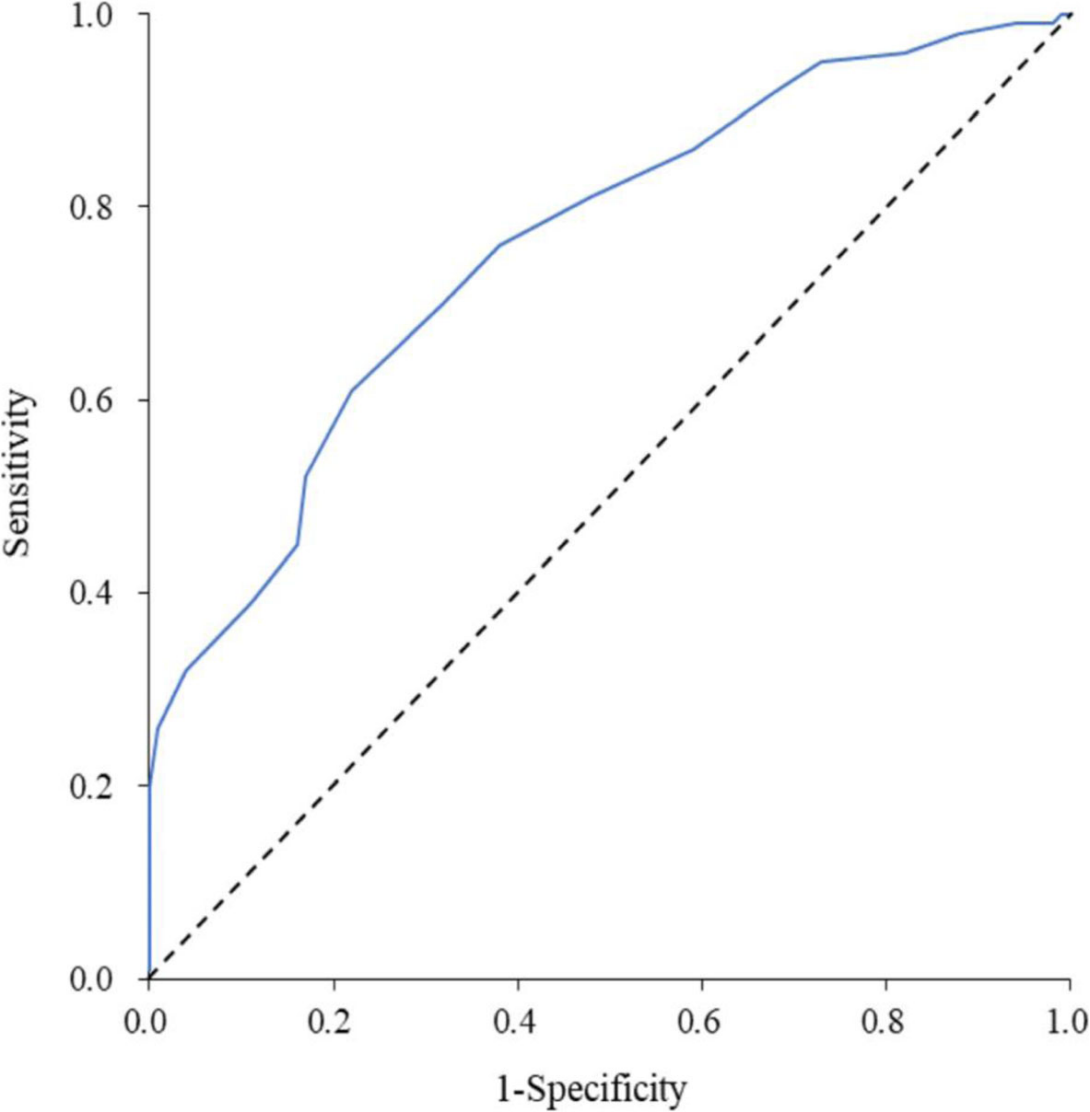
Area under the receiver operating characteristic curve for the scoring system (test set)

The test set was then used to validate and evaluate the scoring model. The test set consisted of 431 patients (330 HFMD and 101 non-HFMD). Within the test set, the median score of the HFMD group was 10 (7, 14) and the median score of the non-HFMD group was 5 (2, 8) (Z = 7.93, *P* < 0.001). The final scores ranged from **−** < 0.001 points with predictive accuracies of 0.12 to 0.99. The AUC was 0.76 (95% CI: 0.710–0.810) (P < 0.001),with a sensitivity of 0.76 and specificity of 0.62. The positive predictive value was 0.87 and the negative predictive value was 0.44 (Fig. 2).

The comparison of general data and exposure history data between the training set and the test set is shown in Table 5. In the test set, the population is divided into three groups according to the tertiles of the predicted probability, and the actual prevalence rate of each group is relatively consistent with the predicted probability of disease (Table 6).

**Table 5:** Comparison of general and epidemiological data between HFMD group and Non-HFMD groups in training set and test set

		Number of patients	Number of male patients	Illness duration (days)	Age (years)	Number of patients with positive exposure history
Training set	HFMD group	764	433	1.48 ± 0.89	1.72 ± 0.76	315
	Non-HFMD group	240	130	1.65 ± 1.01	1.49 ± 0.76	28
	*χ*^*2*^ */ t*		0.47	2.52	4.12	70.97
	*P*		0.495	0.012	<.0001	<.0001
Testing set	HFMD group	330	201	1.50 ± 0.86	1.71 ± 0.77	127
	Non-HFMD group	101	59	1.75 ± 1.02	1.55 ± 0.80	11
	*χ*^*2*^ */ t*		0.2	2.50	1.88	27.05
	*P*		0.654	0.013	<.0001	<.0001

**Table 6:** Comparison of prevalence and predicted probabilities in test set

	Prediction probability range	Score range	Number of patients	Prevalence probability	Predicted probability
Group 1	≤0.6843	−5 ~ 6	143	55.94%	52.82%
Group 2	0.7366 ~ 0.8858	7 ~ 11	147	81.63%	81.30%
Group 3	≥0.9092	12 ~ 24	141	92.20%	94.85%

## Discussion

Accumulating evidence implicates enteroviruses as the most common pathogens associated with acute rash illness in children under three years of age [7] and often manifest as HFMD, affecting the mouth, hands, feet, and buttocks. With increased accuracy and availability of sophisticated laboratory testing, recent studies have found that the distribution of rashes in atypical HFMD differs significantly from that of classic HFMD [8], leading to increased difficulty in making a clinical diagnosis. While definitive diagnosis requires the detection of enterovirus nucleic acids from throat swabs [9], the availability of such technology may be limited in many healthcare settings. In the present study, we analyzed clinical data collected from patients suffering from acute rash illness with confirmatory viral assays to establish an objective, accessible, and sensitive diagnostic scoring system for the rapid identification of HFMD in children under three years of age.

All patients included in this study were children presenting with acute rashes of less than three days in duration. By comparing a large set of clinical data obtained from patient history, physical examination, and routine laboratory tests, we determined the strength of each variable in affecting the accuracy of the final diagnosis. This study demonstrated that older age is predictive of an increased likelihood of HFMD diagnosis, consistent with the established age distribution of the disease [16]. Additionally, the large impact of positive exposure history on diagnostic accuracy supports existing epidemiological findings [17]. Our detailed characterization of rash distribution and density is in agreement with one of the defining features of HFMD, where ulcer/sores of the oral cavity (hard palate, soft palate, and buccal mucosa) have high sensitivity in predicting the illness.

In typical HFMD, rash spots are often presented in the hands and feet (commonly associated with EV71 and CA1 infections), leading to diagnoses being made without using a complex diagnostic scoring system. However, in children presenting with atypical rash distributions, infectious agents can include CV6 [18]. Additionally, EV17 and CV16 are also known to cause atypical rash distribution in young children [19, 20]. The pathogens implicated in our study’s HFMD group were primarily non-EV71 and non-CA16. Thus, the diagnostic scoring system established here is applicable to atypical HFMD. Our study also revealed that examination of the rash severity on the back and the buttocks, regions that may often be overlooked during a clinical encounter, can be critical. We found that rash on the buttocks is more common in children with HFMD while the presence of rash in the back reduces the likelihood of HFMD. For these reasons, clinicians should routinely perform a thorough skin examination in children with acute rash illness to achieve the greatest diagnostic accuracy.

To date, no uniform guidelines have been devised in quantifying rash severity and distribution. Based on the observations from this study, the atypical HFMD rash was qualitatively less fused and flakier, improving discriminability of individual rash spots. Nonetheless, the continued development of objective rash classification is subject to ongoing and future research efforts. For the quantitative assessment of rash covering multiple regions across the body, one method may involve the estimation of the percentage of body surface occupied.

However, the gold standard in the diagnosis of HFMD is the PCR virus-specific nucleic-acid-sequence detection assay, the number of reported cases of HFMD in China in 2018 was 2,353,310 [21] and the vast majority were diagnosed clinically. Laboratory diagnosis of HFMD requires PCR detection, but PCR testing requires clinical gene amplification laboratories, which are not widely accessible China. In 2010, China introduced the “Administrative Measures for Clinical Gene Amplification and Testing Laboratories for Medical Institutions” protocol to improve and increase laboratory infrastructure across the nation. As of January 2014, there were more than 2670 clinical gene amplification testing laboratories that passed national standards. However, this number is still insufficient for China’s large population, and PCR testing is still not available in most remote community health institutions.

Even though HFMD is a self-limiting illness, it is easily transmitted and prone to cause outbreaks in child-care and day-care institutions. Pathogens causing HFMD may lead to large-scale epidemics in a short period of time and can have serious adverse effects on child development. Additionally, the large number of children infected during each outbreak can pose a heavy economic burden on their families and society. Early isolation upon diagnosis is paramount for limiting the spread of HFMD [22], especially in health care facilities and institutions housing medically vulnerable populations. A tool for rapidly diagnosing HFMD with high sensitivity and specificity would improve the efficiency of identifying patients for further PCR testing to confirm diagnosis.

As the clinical scoring systems are designed as a tool to help clinicians make rapid and accurate clinical diagnoses. Our study identified seven clinical variables that impact the accuracy of diagnostic prediction. We defined a score of seven or greater as being suggestive of a clinical diagnosis of HFMD. The diagnostic accuracy of the scoring system was 80% with a sensitivity of 0.76 and a specificity of 0.68, consistent with that of moderate diagnostic performance. All clinical variables of this scoring system may be obtained from clinical history and physical examination without the need for specialized equipment or examination. The scoring scheme is easy to remember and may be utilized across a spectrum of clinical settings. Since the scoring system requires only a rash count, it is cost-effective and can be employed by clinicians in hospitals with limited diagnostic resources.

One limitation of this study is that the applicability of the scoring system has not been validated in a separate cohort or at other institutions. Future multicenter prospective studies may confirm or improve the accuracy of our scoring system. Overall, our scoring system was designed to assist the efficient and accurate diagnoses of acute rash illnesses with the goal of early identification, treatment, and triage of HFMD patients to reduce childhood morbidity and disease transmission.

## Conclusion

In this large retrospective analysis of children with acute rash illness, we identified seven clinical variables with significant impacts on the accuracy of HFMD diagnosis. Due to the systematic and detailed collection of the physical examination data, this study not only confirms existing diagnostic criteria but also emphasizes the importance of examining body regions often ignored during a routine clinical encounter. While future research should focus on validation of this scoring system, its improved diagnostic accuracy is not only limited to typical HFMD but can also extend to atypical presentations of HFMD.

## Data Availability

The data that support the findings of this study are not currently available, as the data also from part of an ongoing study.

## Abbreviations

AUC: Area Under the Receiver Operating Characteristic Curve
CA10: Coxsackievirus group A10
CA16: Coxsackievirus group A16
CA4: Coxsackievirus group A4
CA6: Coxsackievirus group A6
HFMD: Hand-Foot-Mouth Disease
PCR: Polymerase Chain Reaction
ROC: Receiver Operating Characteristic
